# Discovery of New α-Glucosidase Inhibitors: Structure-Based Virtual Screening and Biological Evaluation

**DOI:** 10.3389/fchem.2021.639279

**Published:** 2021-03-08

**Authors:** Shan-Kui Liu, Haifang Hao, Yuan Bian, Yong-Xi Ge, Shengyuan Lu, Hong-Xu Xie, Kai-Ming Wang, Hongrui Tao, Chao Yuan, Juan Zhang, Jie Zhang, Cheng-Shi Jiang, Kongkai Zhu

**Affiliations:** ^1^ School of Biological Science and Technology, University of Jinan, Jinan, China; ^2^ Drug Discovery and Design Center, State Key Laboratory of Drug Research, Shanghai Institute of Materia Medica, Chinese Academy of Sciences, Shanghai, China; ^3^ Zoucheng Administration for Market Regulation, Zoucheng, China; ^4^ Lunan Pharmaceutical Group Corporation, Linyi, China; ^5^Shandong Key Laboratory of Biochemical Analysis, College of Chemistry and Molecular Engineering, Qingdao University of Science and Technology, Qingdao, China

**Keywords:** α-glycosidase, virtual screening, cytotoxicity, type 2 diabetes, molecular docking

## Abstract

α-Glycosidase inhibitors could inhibit the digestion of carbohydrates into glucose and promote glucose conversion, which have been used for the treatment of type 2 diabetes. In the present study, 52 candidates of α-glycosidase inhibitors were selected from commercial Specs compound library based on molecular docking–based virtual screening. Four different scaffold compounds (7, 22, 37, and 44) were identified as α-glycosidase inhibitors with IC_50_ values ranging from 9.99 to 35.19 μM. All these four compounds exerted better inhibitory activities than the positive control (1-deoxynojirimycin, IC_50_ = 52.02 μM). The fluorescence quenching study and kinetic analysis revealed that all these compounds directly bind to α-glycosidase and belonged to the noncompetitive α-glycosidase inhibitors. Then, the binding modes of these four compounds were carefully investigated. Significantly, these four compounds showed nontoxicity (IC_50_ > 100 μM) toward the human normal hepatocyte cell line (LO2), which indicated the potential of developing into novel candidates for type 2 diabetes treatment.

## Introduction

Diabetes is a metabolic disorder that causes high blood sugar and could directly increase the risk of other deadly diseases, such as cancer, stroke, and cardiovascular diseases (Cohen and Goedert, 2004; Zeng et al., 2019). According to the statistics of the World Health Organization (WHO), about 422 million people suffered from diabetes in 2014 around the world, and its prevalence is projected to be 642 million by 2040 (Reusch and Manson, 2017; World Health Organization, 2020). The ineffective use of insulin could result in the type 2 diabetes and accounts for more than 90% of diabetes cases (Proença et al., 2017).

**FIGURE 1:**
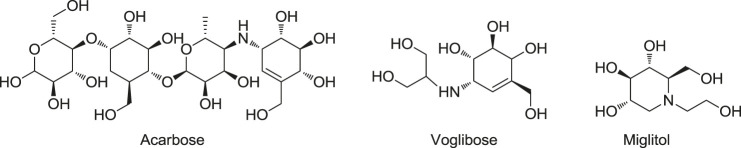
Clinically Approved α-glucosidase inhibitors.

Controlling blood glucose levels is thought to be the main strategy for treating diabetes and reducing diabetes complications (Ye et al., 2019). α-Glucosidase is a key carbohydrate hydrolase that regulates blood glucose by specifically hydrolyzing 1,4-α-glucopyranosidic bond to produce α-glucose (Kazmi et al., 2018). Early studies have shown that the inhibition of α-glucosidase activity could retard the absorption of glucose and decrease the postprandial blood glucose levels (Park et al., 2008; Kim et al., 2019). Therefore, α-glucosidase has been taken as a key target for treating diabetes, and the inhibitors of α-glucosidase can be developed into effective therapeutic drugs to treat this disease (Li et al., 2010). α-Glucosidase inhibitors such as acarbose, miglitol, and voglibose (shown in Figure 1) are the most well-known ones (Joshi et al., 2015). Acarbose, the first approved drug in α-glucosidase inhibitor category, was used to delay the release of glucose from polysaccharides by binding with α-glucosidase. Voglibose was used to discontinue the uptake and hydrolysis of saccharides by selectively inhibiting α-glucosidase vs. pancreatic α-amylase and lactase. Miglitol, the first pseudo-monosaccharide α-glucosidase inhibitor, was approved to reduce postprandial glucose (Hossain et al., 2020). However, some unexpected adverse effects (for instance, flatulence, diarrhea, and stomachache) limited their clinical application. Based on this background, numerous efforts have been carried out to discover new a-glucosidase inhibitors from diverse sources, such as natural products and chemical synthetic compounds (Chen et al., 2017; Liu and Ma, 2017; Abbas et al., 2019; Dhameja and Gupta, 2019).

Virtual screening has been proven to be a very effective tool capable of providing drug hits or leads with structural diversity and makes drug discovery faster and more efficient (Kitchen et al., 2004; Kontoyianni, 2017). In this study, molecular docking–based virtual screening on Specs database was conducted to identify α-glucosidase inhibitors with new chemotypes. After testing the purchased 52 compounds that were obtained by docking screening, four compounds, namely, 7, 22, 37, and 44 with different scaffolds, were disclosed as new α-glycosidase inhibitors. Kinetic analysis of these compounds revealed that they inhibited α-glycosidase activity in a noncompetitive type. Then, the binding modes of these compounds with α-glycosidase were investigated, and the results indicated that all of these compounds could be well located in the acarbose-binding site and displayed very similar binding poses. Moreover, the cytotoxicity of these compounds toward the human normal hepatocyte cell line (LO2) was evaluated. The present results provided new α-glycosidase inhibitors serving as hit compounds for developing novel medications used in the treatment of type 2 diabetes.

## Methods and Materials

### Molecular Docking–Based Virtual Screening

The protein coordinates in the α-glycosidase crystal complex structure (PDB code 3W37) were prepared by the Protein Preparation Wizard panel inserted in the Maestro with the default settings. Residues within 15 Å centered on acarbose were defined as compound-binding sites in which the docking grid was generated by the Receptor Grid Generation panel. The default settings were adopted for the cutoff, neutralization, etc. The docked compounds in Specs database were prepared with LigPrep panel. Then, the prepared compounds were docked to the aforementioned docking gird with extra precision (XP) mode. “Clustering Molecules” protocol inserted in Pipeline Pilot 7.5 was employed to achieve the cluster analysis. The top ranked compounds assessed by XP GScore were clustered into 30 clusters. To increase the diversity of selected compounds, at least one candidate was selected in each cluster. In addition, we gave priority to the compounds with simple structure and/or small molecular weight.

### α-Glycosidase Inhibitory Assay

The α-glucosidase inhibitory evaluation of the purchased 52 compounds was performed according to the previously described protocol (Tang et al., 2014; Ye et al., 2019). α-Glucosidase (Sigma, G5003) derived from baker’s yeast, and pNPG (Sigma, N1377) and the substrate were both purchased from Sigma-Aldrich. 1-Deoxynojirimycin was used as the positive control. The tested compounds and 1-deoxynojirimycin were dissolved in DMSO, the α-glucosidase and the substrate pNPG were both dissolved in phosphate buffer (pH = 6.8). The compounds and α-glucosidase were preincubated in phosphate buffer (37°C, 15 min). Then, 25 μL substrate buffer was added to the system to start the reaction, and the incubation was continued at 37°C for 15 min. Finally, the reaction was terminated by the addition of 50 μL 0.2 M reaction termination solution. The optical density (OD) was measured at an absorbance wavelength of 405 nm using a microplate reader (Tecan, Switzerland). The IC_50_ values were estimated with six different concentrations, and each sample was measured three times in parallel experiments.

### Fluorescence Quenching Experiment

According to the previously reported method (Aguilar-Moncayo et al., 2010), all fluorescence spectra were measured on a fluorescence spectrophotometer (Agilent Cary Eclipse) equipped with a 10.0-mm quartz cell and a thermostat bath. In the fluorescence spectrophotometer, α-glucosidase (1 U/ml) was pretreated with certain concentrations of inhibitors for 30 min at 37°C. 100 μL of the above solution (pH 6.8) was added accurately to the quartz cell. The blank was used for buffer spectrum values. The fluorescence emission spectra were measured at 37°C. The excitation wavelength was 290 nm, and the emission spectrum was recorded from 320 to 500 nm.

### Kinetic Assay

The inhibition type of the inhibitors against α-glucosidase activities was evaluated based on a described method (Hou et al., 2009). Increasing concentrations of substrates pNPG were used in the absence or presence of tested compounds at four different concentrations around the IC_50_ values. The inhibitory kinetics of the investigated compounds on α-glucosidase was analyzed using the Lineweaver–Burk plot of the substrate concentration and velocity.

### Cell Viability Assay

The LO2 cell line was cultured in a proper medium supplemented with 10% fetal bovine serum in a humidified atmosphere of 5% CO_2_ at 37°C. Cell suspensions were plated in 96-well plates at a density of 2 × 10^4^ cells/cm^3^. Compounds were solubilized in DMSO at six different concentrations. After incubation for 24 h, the cells were treated with various concentrations of tested substances for 48 h and then incubated with 100 μL of MTT at 37°C for 2 h. The formazan dye product was measured by the absorbance at 490 nm on a Tecan Spark multimode microplate reader (Switzerland).

## Results and Discussions

### Fifty-Two Candidates of α-Glycosidase Inhibitor Were Selected From the Molecular Docking–Based Virtual Screening Result

As the crystal structure of α-glycosidase–acarbose complex has been determined (PDB code 3W37) (Tagami et al., 2013), molecular docking–based virtual screening could be performed. Specs database that contains 200,000 compounds was chosen as the screening database. The redock result of acarbose (Supplementary Figure S1) declared that GLIDE program (Halgren et al., 2004) inserted in the Schrödinger program suite could well reproduced the binding mode of acarbose in the crystal structure. The top 300 molecules ranked by the docking score were selected for the following cluster analysis. Finally, 52 compounds were retained and purchased from the Specs database supplier for further α-glycosidase enzymatic inhibition activity evaluation.

### *In vitro* Inhibition Test Against α-Glycosidase Identified Four Active Compounds 7, 22, 37, and 44

**FIGURE 2:**
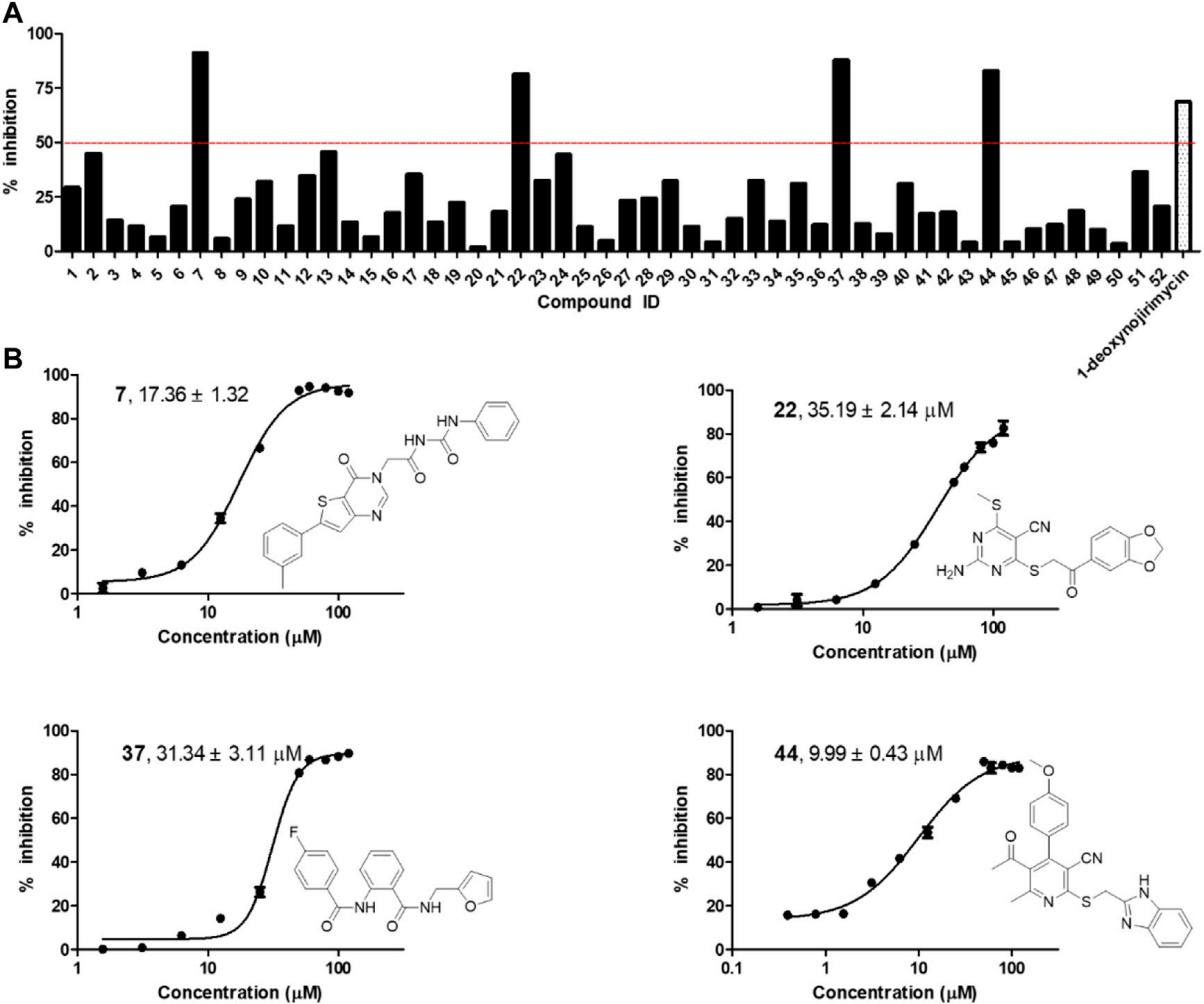
**(A)** α-glycosidase inhibitory activity of the 52 selected candidates at 100 μM; **(B)** The chemical structures and IC_50_ curves of compounds 7, 22, 37, and 44. IC_50_ data are shown as mean ± SD of three independent experiments.

The selected 52 candidates were initially evaluated for their inhibitory ratios against α-glycosidase at 100 μM with 1-deoxynojirimycin as positive reference. The α-glycosidase enzymatic inhibition bioassay results indicated that four compounds, namely, 7, 22, 37, and 44 with representing totally different scaffolds, exhibited an inhibition ratio above 50% at 100 μM (Figure 2A). Then, the IC_50_ values of these four compounds were further determined. As shown in Figure 2B, compounds 7, 22, 37, and 44 displayed IC_50_ values of 17.36 ± 1.32, 35.19 ± 2.14, 31.34 ± 3.11, and 9.99 ± 0.43 μM, respectively. All of them showed better activity than the positive reference control 1-deoxynojirimycin (IC_50_, 52.02 ± 3.78 μM), and compound 44 exhibited the most potent activity.

### Fluorescence Quenching Assay Confirmed the Binding of 7, 22, 37, and 44 to α-Glycosidase

**FIGURE 3:**
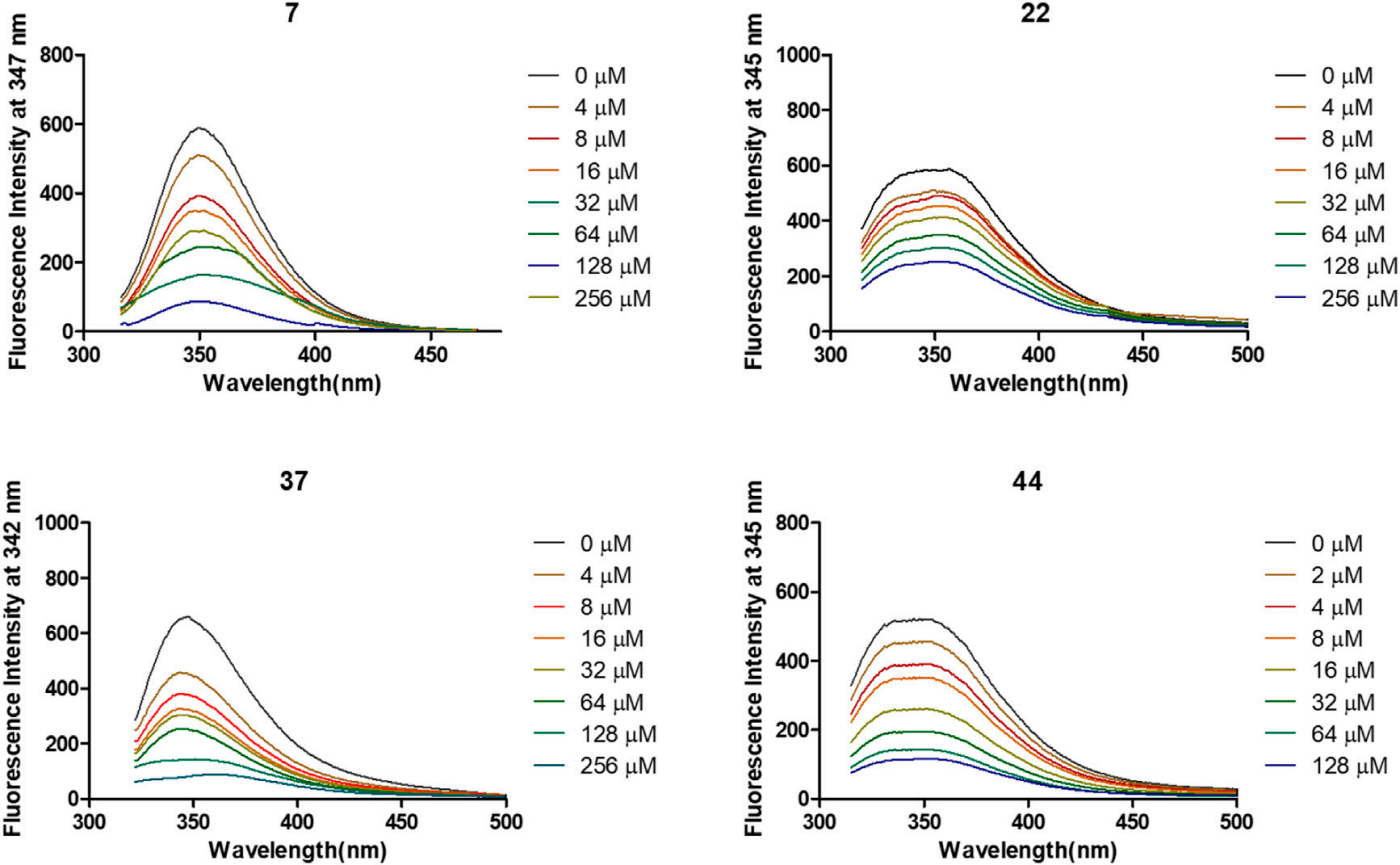
Variation of fluorescence emission spectra of α-glycosidase (1 U/ml) in the presence of compounds 7, 22, 37, and 44 with increasing concentration for 30 min at 37°C.

The interactions of 7, 22, 37, and 44 with α-glycosidase were explored through the fluorescence quenching experiments. As displayed in Figure 3, the variations of the intrinsic fluorescence emission of α-glycosidase (2 μM) in the presence of increasing concentration of molecules 7, 22, 37, and 44, respectively, were recorded at 37°C with the wavelength range from 320 to 500 nm. The intrinsic fluorescence emission peak at 345 nm was observed after being excited at 290 nm.

**FIGURE 4:**
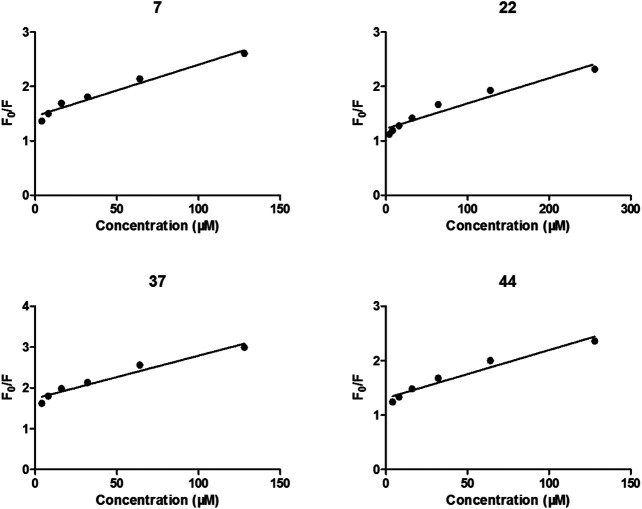
Stern-Volmer plots for the fluorescence quenching of α-glycosidase by compounds 7, 22, 37, and 44.

After treated by compounds 7, 22, 37, and 44 with increasing concentration (Figure 4), the fluorescence intensities of enzyme in all tested systems were gradually quenched in a type of concentration-dependent manner. Thus, these results confirmed the binding of these inhibitors to α-glycosidase.

### Kinetic Study on α-Glycosidase Inhibition Declared the Noncompetitive Manner of These Four Compounds

**FIGURE 5:**
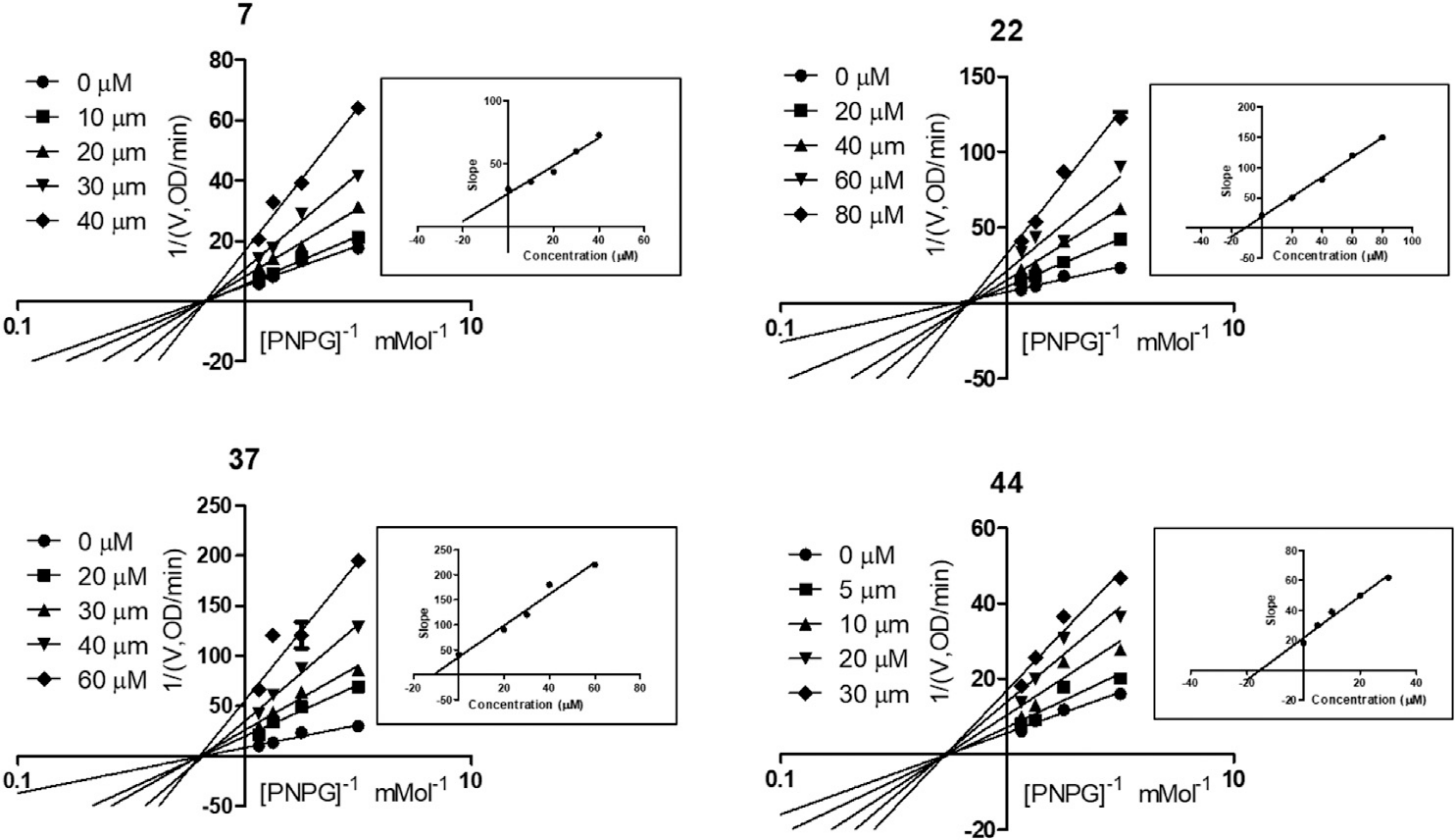
Kinetic assay on α-glycosidase inhibition by compounds 7, 22, 37, and 44, respectively. Lineweaver-Burk reciprocal plots of initial velocity and increasing substrate (PNPG) concentration with secondary plot of slopes vs. the concentration of compounds.

To explore the mechanism of the interaction modes of compounds 7, 22, 37, and 44 with the enzyme, kinetic assay was conducted to study their inhibition types using Lineweaver–Burk plot analysis (Wang et al., 2004; Sun et al., 2018). The results shown in Figure 5 indicate that compounds 7, 22, 37, and 44 were noncompetitive α-glycosidase inhibitors, with estimated *K*_i_ values of 24.18, 11.34, 11.27, and 15.39 μM, respectively.

### Molecular Docking Simulation Revealed the Binding Mode of These Four Compounds

**FIGURE 6:**
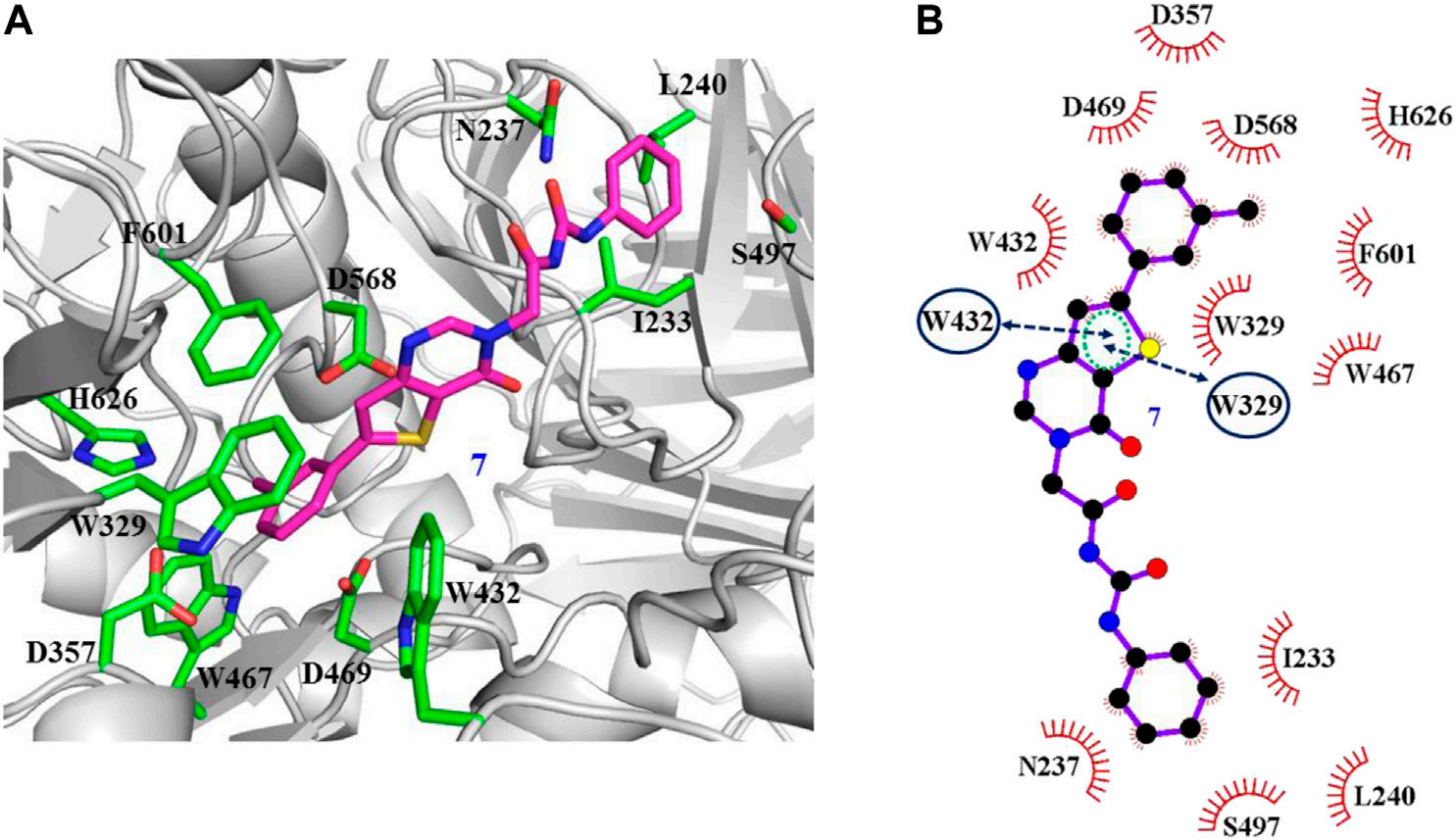
Docking pose of compound 7 bound to the acarbose binding site in α-glycosidase. **(A)** The three-dimensional interacting modes between 7 and α-glycosidase. α-Glycosidase, 7 and the interacting residues were shown as cartoon, sticks (carbon atoms colored in magenta), sticks (carbon atom colored in green), respectively. **(B)** Schematic representation displayed the hydrophobic interactions (shown as starbursts) and Pi-Pi interactions (shown as oval) of 7 with α-Glycosidase.

**FIGURE 7:**
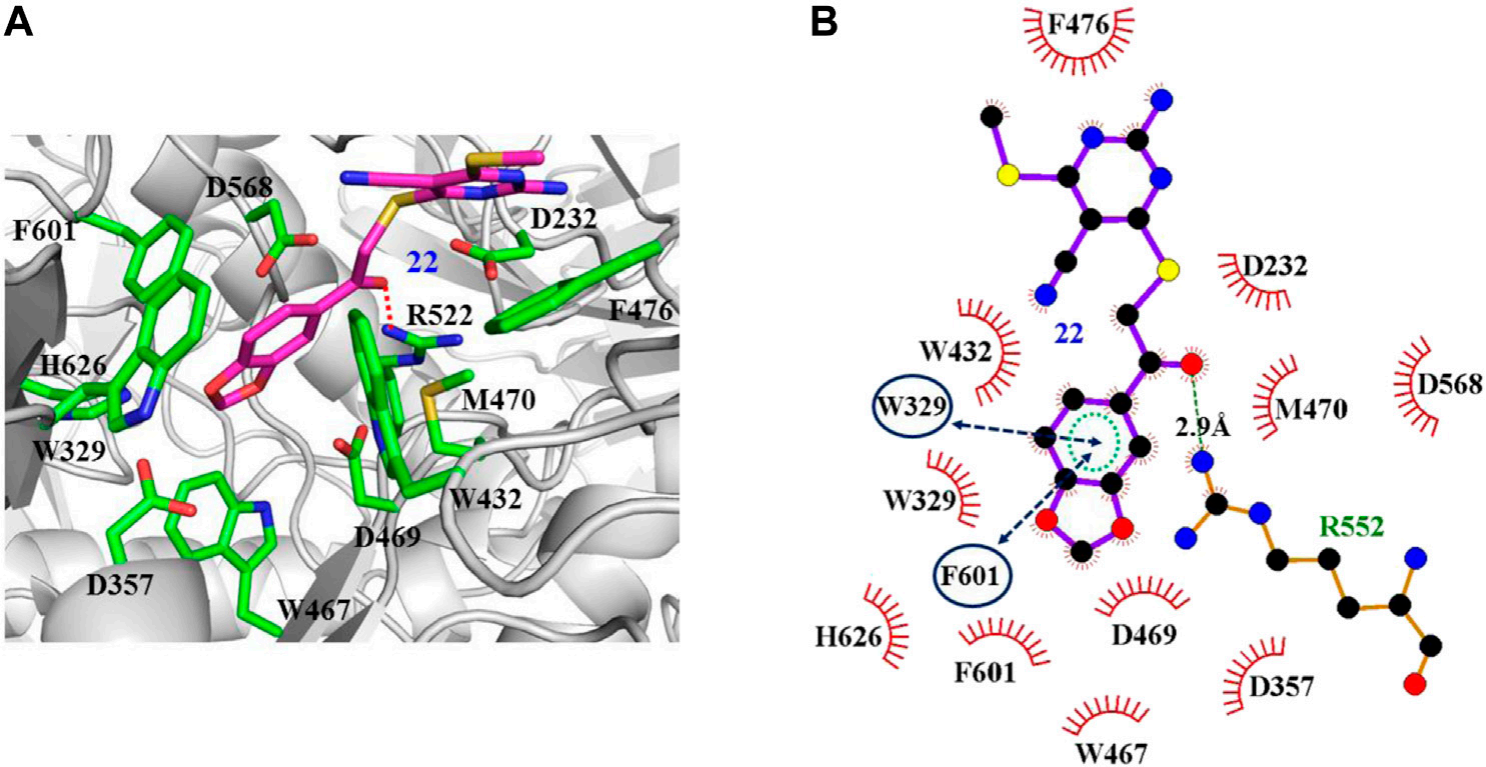
Docking pose of compound 22 bound to the acarbose binding site in α-glycosidase. **(A)** The three-dimensional interacting modes between 22 and α-glycosidase. α-Glycosidase, 22 and the interacting residues were shown as cartoon, sticks (carbon atoms colored in magenta), sticks (carbon atom colored in green), respectively. **(B)** Schematic representation displayed the hydrophobic interactions (shown as starbursts), Pi-Pi interactions (shown as oval), and H-bond interactions (denoted by dotted green lines) of 22 with α-Glycosidase.

**FIGURE 8:**
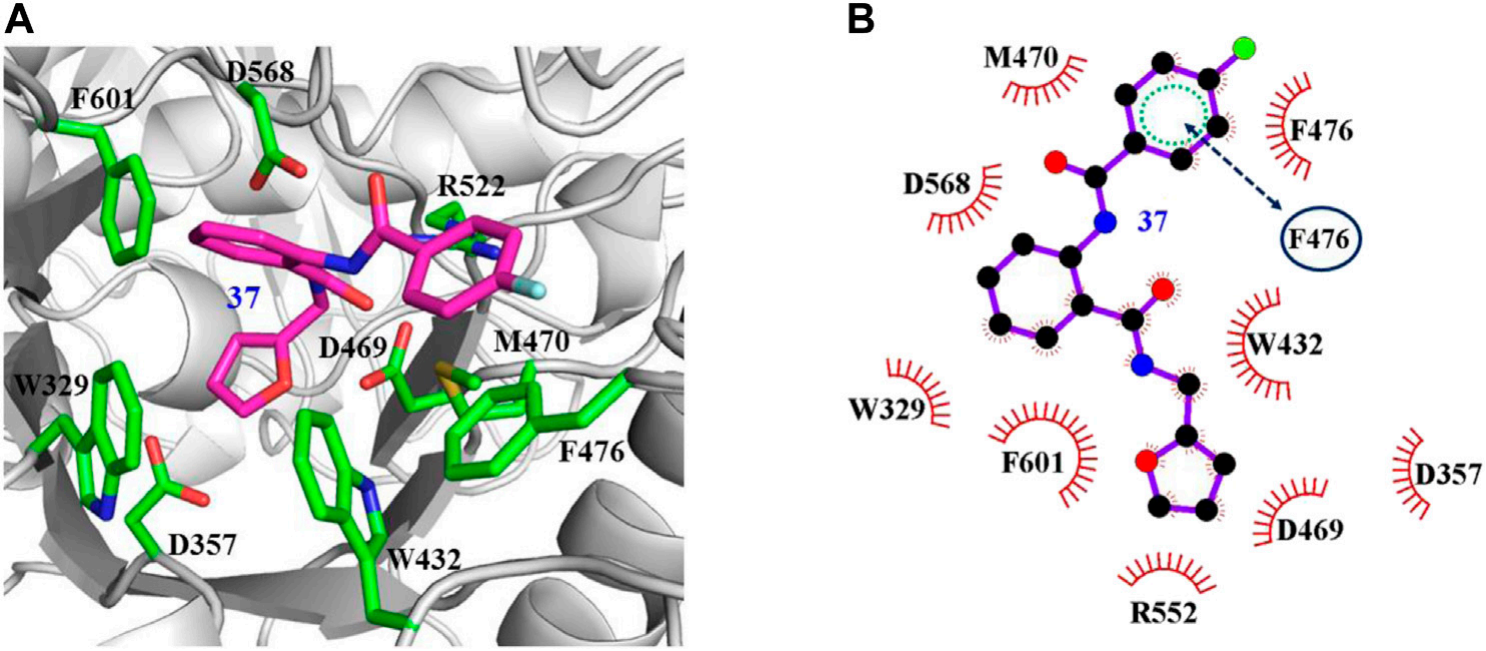
Docking pose of compound 37 bound to the acarbose binding site in α-glycosidase. **(A)** The three-dimensional interacting modes between 37 and α-glycosidase. α-Glycosidase, 37 and the interacting residues were shown as cartoon, sticks (carbon atoms colored in magenta), sticks (carbon atom colored in green), respectively. **(B)** Schematic representation displayed the hydrophobic interactions (shown as starbursts) of 37 with α-Glycosidase.

**FIGURE 9:**
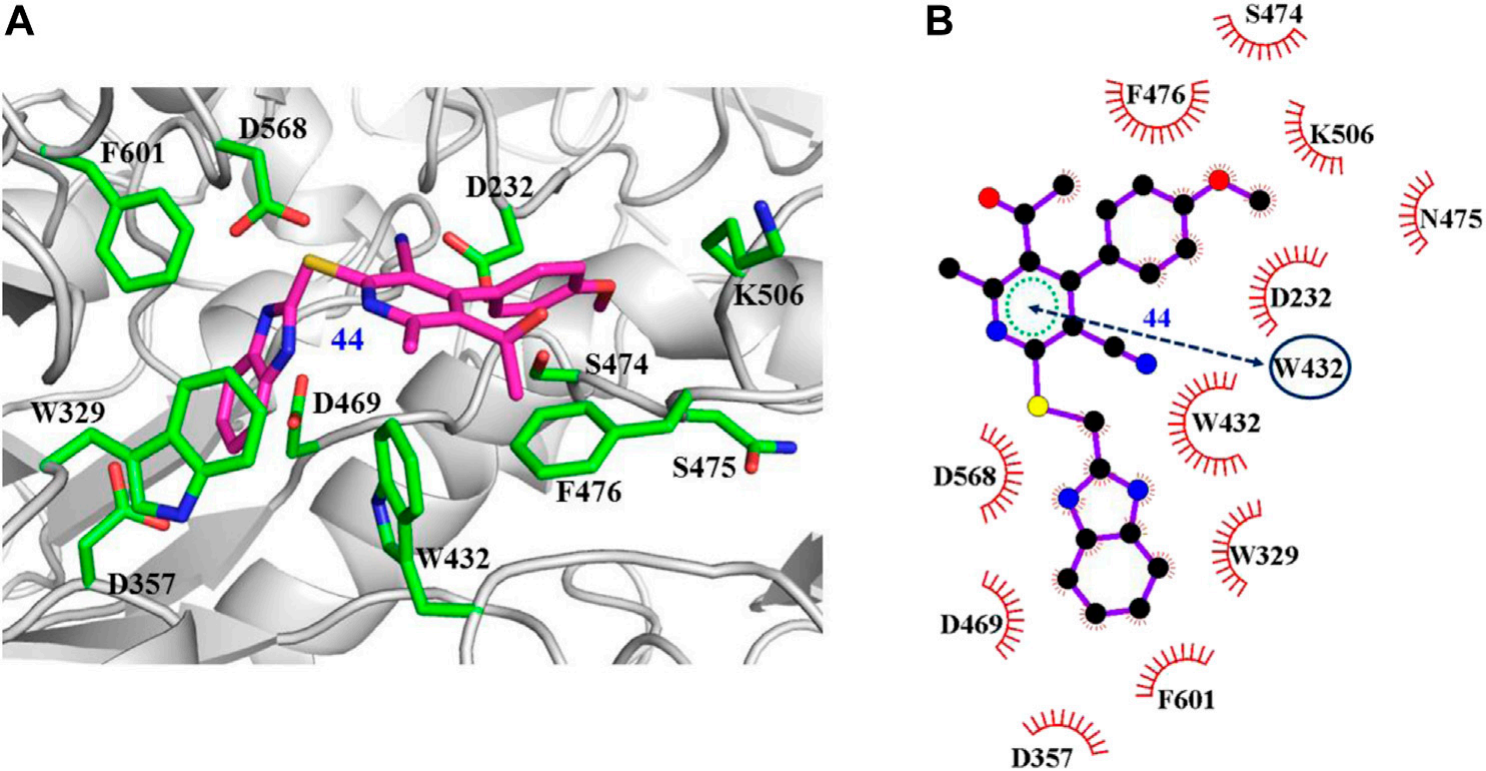
Docking pose of compound 44 bound to the acarbose binding site in α-glycosidase. **(A)** The three-dimensional interacting modes between 44 and α-glycosidase. Compound 44 and the interacting residues were shown as cartoon, sticks (carbon atoms colored in magenta), sticks (carbon atom colored in green), respectively. **(B)** Schematic representation displayed the hydrophobic interactions (shown as starbursts) and Pi-Pi interactions (shown as oval) of 44 with α-Glycosidase.

The interaction mechanisms of compounds 7, 22, 37, and 44 with α-glycosidase were carefully analyzed with the molecular docking results, as shown in Figures 6–9. All these four compounds could well bind to the allosteric sites away from the active site (Asp214, Glu276, and Asp349) (Ye et al., 2019) in α-glycosidase and formed hydrophobic interactions with nearby residues. These results were consistent with the noncompetitive property. The docking scores of these four hits were -3.811, -2.825, -3.627, and -6.283. Specifically, inhibitor 7 established hydrophobic interactions with residues D357, D469, W432, N237, S497, L240, I233, W329, W467, F601, H626, and D568 and formed Pi–Pi stacking with residues W329 and W432. Compound 22 formed hydrophobic interactions with residues W329, F476, D357, D469, W432, D232, M470, W467, F601, H626, and D568 and formed Pi–Pi stacking with residues W329 and F601. Besides, 22 established H bond interaction with R552 residue. Compound 37 formed hydrophobic interaction with residues M470, W329, F476, D357, D469, W432, F601, D568, and R552 and formed Pi–Pi stacking with residue F476. Additionally, compound 44 formed hydrophobic interaction with residues S474, W329, F476, D357, D469, W432, N475, D568, D232, F601, and K506 and formed Pi–Pi stacking with residue W432. From these data, we could find that residues W432, W329, F601, D357, D469, and D568 were the key residues contributing interaction with all of the four compounds.

### *In vitro* Cytotoxicity

Since most of the drugs are metabolized in the liver, there is a great focus on the hepatic safety of new medicines. Thus, the cytotoxicity of inhibitors 7, 22, 37, and 44 was evaluated in human normal hepatocyte (LO2) cells using the MTT method (Ge et al., 2020). The results disclosed that all of these compounds had IC_50_ values more than 100 μM toward LO2 cells, suggesting they are nontoxic toward liver cells. Thus, further structural optimization and biological evaluation for 7, 22, 37, and 44 deserved further investigation.

## Conclusion

In this study, four novel α-glycosidase inhibitors 7, 22, 37, and 44 with distinct structural features were identified through virtual screening and *in vitro* evaluation. Among them, compound 44 had the best α-glycosidase inhibitory activity with IC_50_ and *K*_i_ values of 9.99 ± 0.43 and 15.39 μM, respectively. The fluorescence quenching experiment indicated all these compounds could directly bind to α-glycosidase, and the kinetic study revealed a noncompetitive α-glycosidase inhibitory mechanism of these compounds toward α-glycosidase. In addition, binding mode analysis provided the detailed binding mechanism of these four α-glycosidase inhibitors, which made further structural optimization feasible. Moreover, the *in vitro* cytotoxicity bioassay demonstrated these α-glycosidase inhibitors were nontoxic toward LO2 cells. Based on these results, these compounds can serve as promising hit compounds for further bioactivity optimization and anti–type 2 diabetes study.

## Data Availability

The original contributions presented in the study are included in the article/Supplementary Material, further inquiries can be directed to the corresponding authors.
